# A Rare Case of COVID-19 Myocarditis With Cardiac Tamponade in a Young Diabetic Adult With Renal Failure

**DOI:** 10.7759/cureus.11632

**Published:** 2020-11-22

**Authors:** Tarkeshwar Tiwary, Shalanki Baiswar, Praveen Jinnur

**Affiliations:** 1 Pulmonary Medicine, WellSpan Health, Chambersburg, USA; 2 Internal Medicine, WellSpan Health, Chambersburg, USA; 3 Internal Medicine, Essentia Health, Fargo, USA

**Keywords:** covid 19, pericardial tamponde, covid and myocarditis

## Abstract

A young male with long-standing type 1 diabetes mellitus, chronic kidney disease, and known ventricular hypertrophy presented with dyspnea and abdominal pain and was diagnosed with coronavirus disease 2019 (COVID-19) infection. On day nine of hospital admission, patient developed ventricular tachycardia with electrocardiogram (ECG) changes and elevation in troponin level consistent with myocarditis and development of cardiogenic shock. Bedside limited echo demonstrated signs of tamponade and patient underwent surgical pericardial window procedure. He was also noted to develop marked prolongation of corrected QT interval (QTc) while on amiodarone.

## Introduction

Coronavirus disease 2019 (COVID-19)-induced myocarditis has been noted to be a relatively late complication in the natural history of the disease. Pericardial effusion with tamponade physiology in this setting has been infrequently reported [1,2]. The risk of cardiac tamponade may be particularly high in patients with concomitant renal failure and more severe immune dysregulation in the setting of multi-organ failure [3].

## Case presentation

A 30-year-old Latino male with a history of type 1 diabetes for the past 16 years, diabetic nephropathy with chronic kidney disease (CKD) stage III, glaucoma, hypertension, and obesity presented to the emergency department with bilateral abdominal flank pain and shortness of breath. The patient additionally had symptoms of fatigue, tiredness, and lightheadedness. The patient did not report any fever, hemoptysis, chest pain, palpitation, or syncope at presentation.

**Figure 1:**
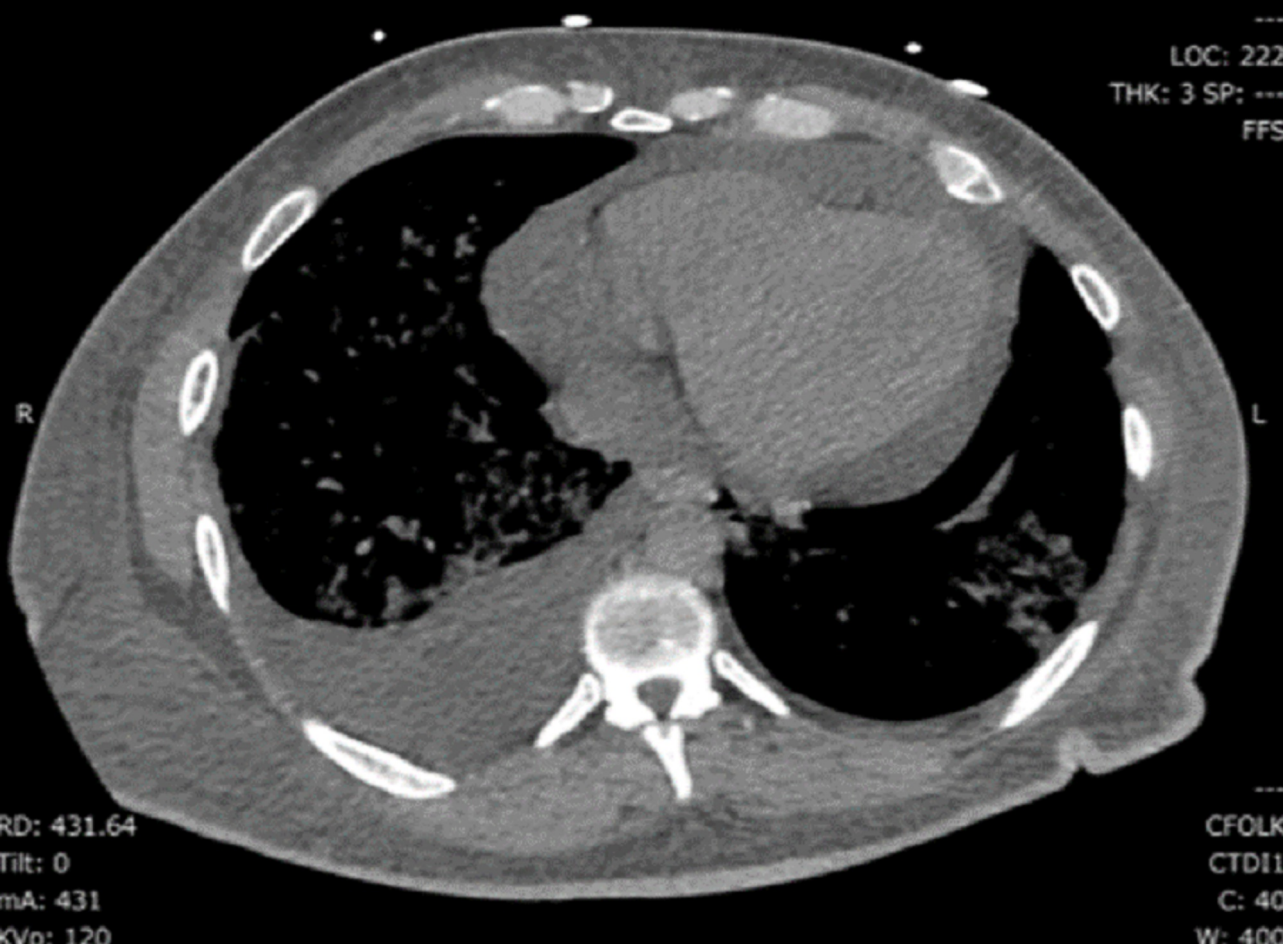
Computed tomography of the chest. Mediastinal window showing pericardial and pleural effusion (Day 3)

**Figure 2:**
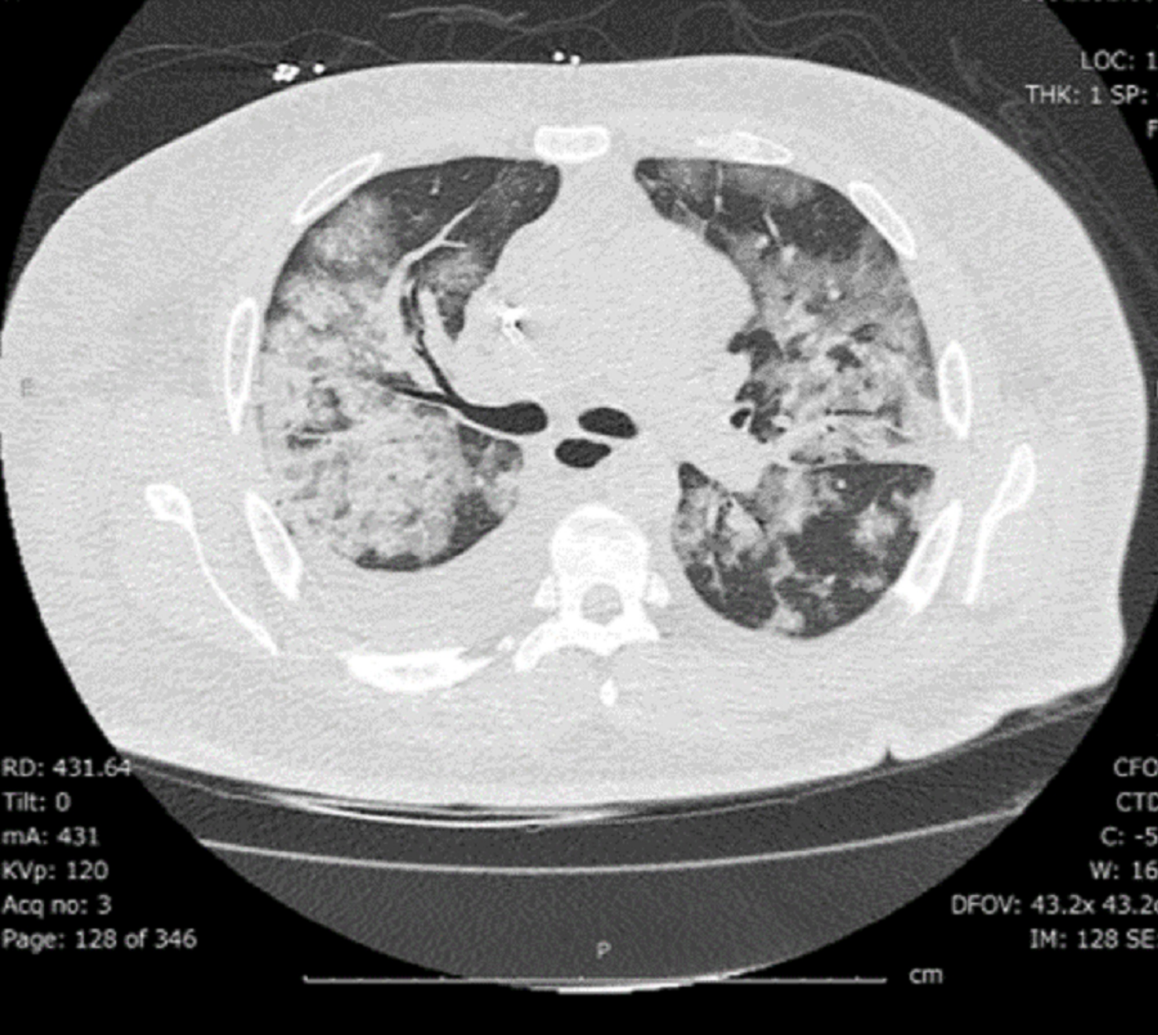
Computed tomography of the chest. Lung window showing typical B/L consolidative changes superimposed on ground glass changes (Day 3)

**Figure 3:**
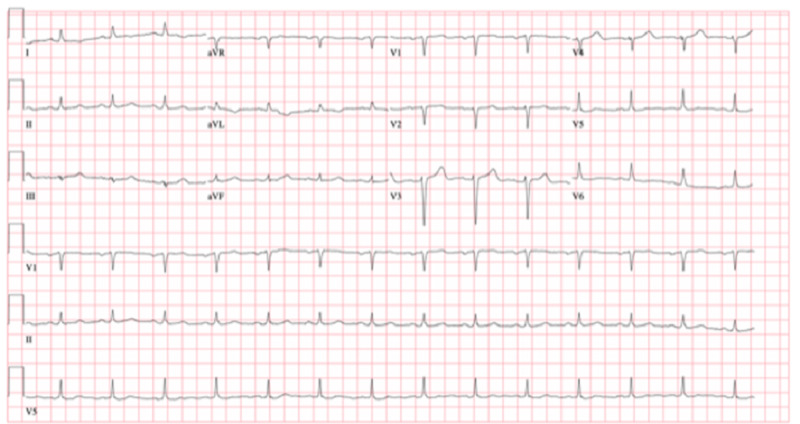
Baseline electrocardiogram on admission: QRS-92 ms, QT/QTc-388/447 ms, PR 170 ms

Reverse transcription-polymerase chain reaction (RT-PCR) for severe acute respiratory syndrome coronavirus 2 (SARS-CoV-2) was positive on the day of admission. On admission, laboratory test values were brain natriuretic peptide of 557 pg/mL, creatinine of 4.74 mg/dL, blood urea nitrogen of 63 mg/dL, white blood cell count of 5.4 K/UL, hemoglobin of 6.6 g/dL, and platelet count of 220 K/UL. The liver function test result was normal, and ferritin was 218 ng/mL which increased to 1346 ng/mL on the 10th day after admission. C-reactive protein was 89 mg/L on the second day after admission and increased to 259 mg/L on the 10th day after admission. Troponin I was 0.09 ng/mL on admission, then increased to 7.52 ng/mL on the 10th day after admission and then decreased to 2.57 on day 12. The gene Xpert RT-PCR test for SARS-CoV-2 was positive 24 days after the initial diagnosis. The partial thromboplastin time was persistently elevated less than two times the upper limit of normal, and D-dimer was significantly elevated to 10 times that of normal on day 11. Blood cultures and sputum culture screens were negative, and procalcitonin was not elevated. The initial computed tomography of the chest on day three showed typical changes consistent with COVID-19 pneumonia with right pleural effusion and pericardial effusion (Figure 1, 2). The baseline electrocardiogram (ECG) on presentation is shown in Figure 3.

**Figure 4:**
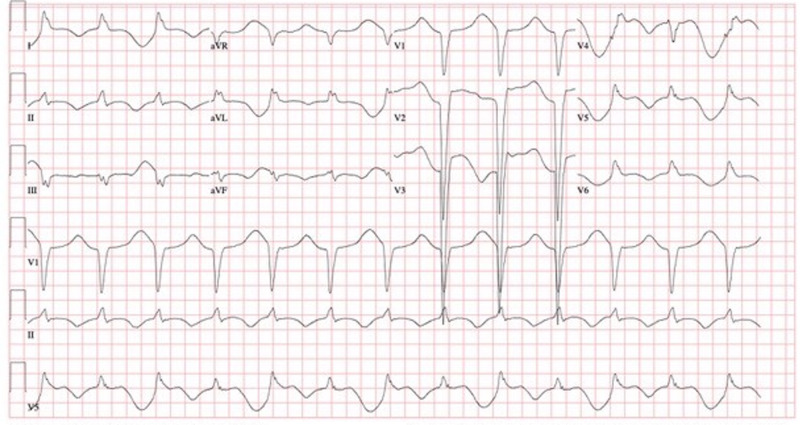
Day nine pre-pericardiotomy – QRS-166 ms, QT/QTc-796/853 ms, and accelerated idioventricular rhythm with IV conduction block

The patient initially improved on supplemental oxygen with a nasal cannula at 2 L/min. On the fourth day after admission, his fraction of inspired oxygen (FiO2) requirement continued to progressively increase over 12 hours to 100% supplemental oxygen by a high-flow nasal cannula system. The patient was switched to noninvasive positive pressure ventilation, but he continued to require an FiO2 of 80%. Later on the same day, the patient developed respiratory distress and was subsequently intubated and started on mechanical ventilatory support per the Acute Respiratory Distress Syndrome Network trial protocol. Continuous renal replacement therapy was also initiated for the patient for acute on chronic renal failure with volume overload. By day nine of hospitalization, the patient's oxygenation status had improved down to 60% FiO2, but during the evening of the same day, he developed episodes of hypotension, and norepinephrine and vasopressin infusion for vasopressor support were initiated. The patient also had episodes of non-sustained ventricular tachycardia. Twelve-lead ECG showed a wide QRS complex along with new-onset left bundle branch block (Figure 4).

**Figure 5:**
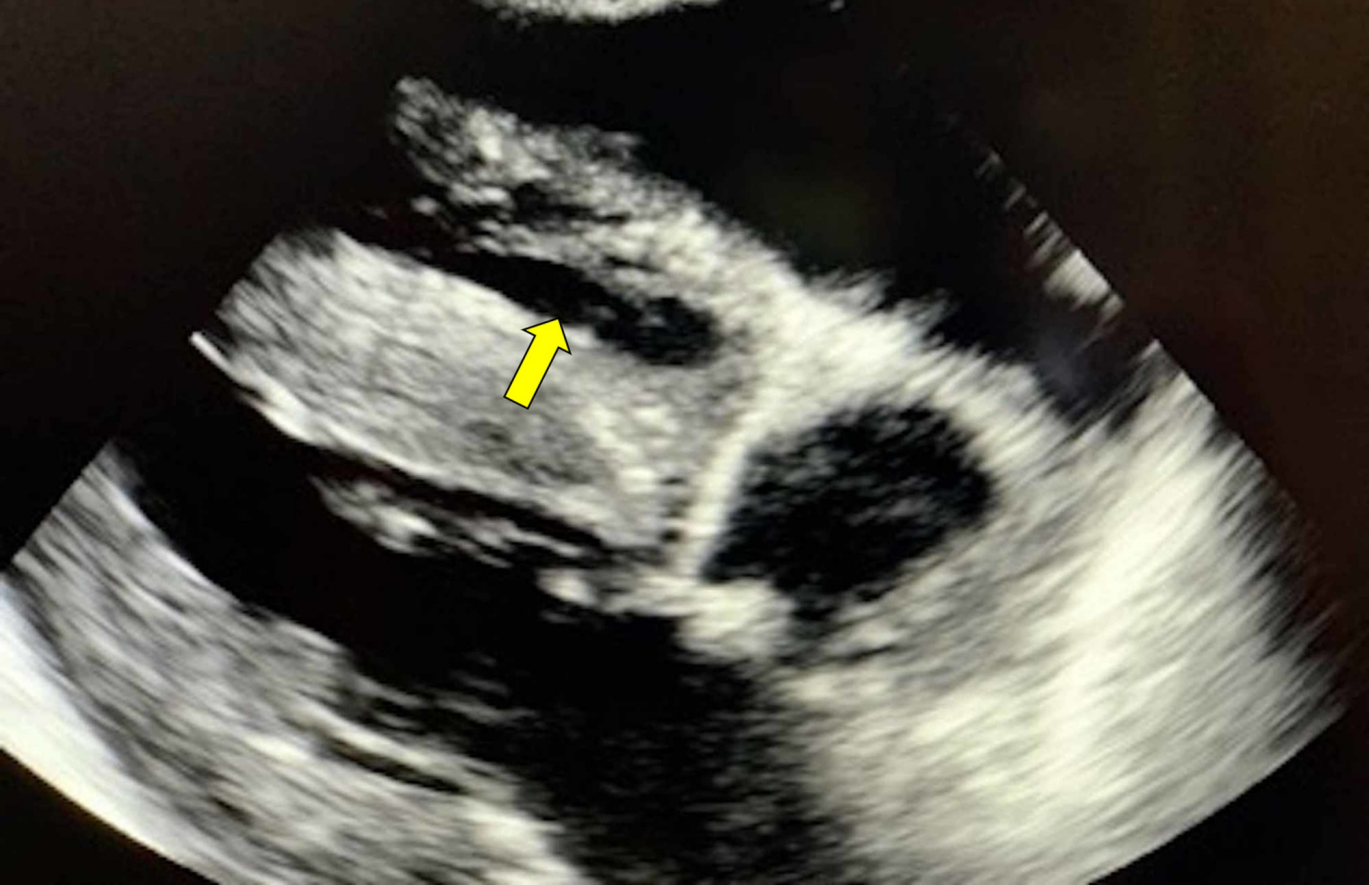
Point of care echocardiogram with parasternal window long-axis (PLAX)-modified view showing severe left ventricular hypertrophy and diastolic right ventricular collapse (arrow indicates collapsing right ventricle in diastole).

Amiodarone infusion was initiated for high risk of malignant arrhythmia. Bedside echocardiography was performed, and it showed large pericardial effusion with early diastolic right ventricle prolapse along with a markedly thickened ventricular wall (Figure 5).

**Figure 6:**
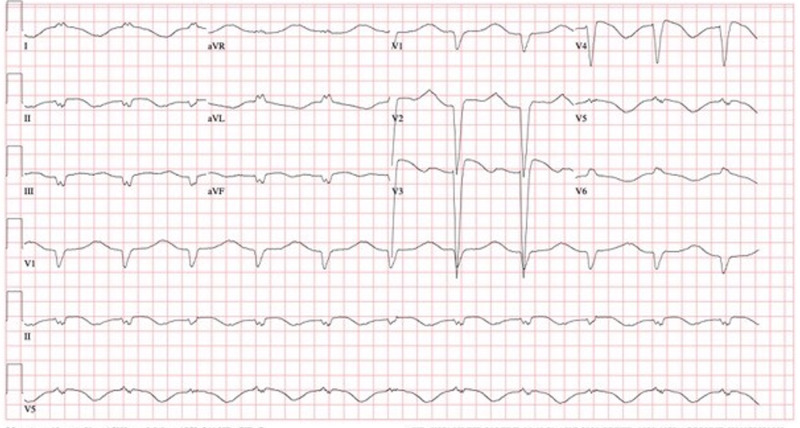
Day nine post-pericardiotomy, QRS-168 ms, QT/QTc 752/788 ms

The patient was examined in January 2020, and at that time, an echocardiogram showed moderate ventricular hypertrophy along with trivial pericardial effusion. Based on these past results and the current situation, the patient underwent a surgical pericardial window procedure. Amiodarone was discontinued due to marked elevation in the corrected QT interval (QTc) to 853 ms (Figure 6). The patient was treated with remdesivir, convalescent plasma, and dexamethasone and receieved empirical antibacterial coverage with cefepime and doxycycline. Patient did not receive hydroxychloroquine or azithromycin during hospitalization.

Improvement in hemodynamics post-procedure was noted, with discontinuation of vasopressor support on the same day. The patient was extubated on day five postpericardiotomy after 15 days of mechanical ventilator support. The patient was discharged home on day 27 of hospitalization. Before discharge, he was hemodynamically stable without any ECG abnormality, had normal left ventricular systolic function, and trivial pericardial effusion on two-dimensional echocardiography and was oxygenating well on room air.

## Discussion

Acute respiratory distress syndrome is the most common indication for transferring patients with COVID-19 to the intensive care unit and the primary cause of death in this patient population. Cardiac involvement with myopericarditis is a relatively late phenomenon in the disease progression but is suspected to be the primary reason for sudden cardiac death in patients with COVID-19 [3,4]. A few pericardial effusion cases with tamponade physiology in COVID-19 patients have been previously described [5-8].

Six months before the current hospital admission, our patient exhibited ventricular hypertrophy and trivial pericardial effusion at baseline echocardiography. Due to the development of acute kidney injury (AKI) on chronic kidney disease at presentation, the patient was hypervolemic, which led to pleural effusion and worsening of pericardial effusion compared to baseline echo. On approximately day 10 after presentation, our patient developed myopericarditis features with conduction abnormalities, QRS prolongation, non-sustained ventricular tachycardia, and elevation in troponin disproportionate to AKI. The patient also developed cardiogenic shock requiring vasopressor support. Bedside point-of-care echocardiography was diagnostic of early pericardial tamponade with significant systolic function impairment due to decreased cardiac filling. Post-pericardiotomy, vasopressor support was slowly withdrawn over 24 hours, and the ventilator was no longer necessary on postoperative day five.

## Conclusions

Pericardial tamponade should be strongly considered in the differential diagnosis of acute hypotension with signs of myocarditis in COVID-19 patients, particularly with acute renal failure. Due to the variable and limited availability of standard echocardiography for patients in airborne isolation because of infection control concerns, bedside point-of-care echocardiography is very useful in determining a differential diagnosis of hemodynamic instability in critically ill patients. Marked prolongation of QTc was noted in our patient on amiodarone, which suggests the need for close monitoring of QTc in patients with myocarditis who are receiving QTc-prolonging medications.
